# Physical Realization of a Supervised Learning System Built with Organic Memristive Synapses

**DOI:** 10.1038/srep31932

**Published:** 2016-09-07

**Authors:** Yu-Pu Lin, Christopher H. Bennett, Théo Cabaret, Damir Vodenicarevic, Djaafar Chabi, Damien Querlioz, Bruno Jousselme, Vincent Derycke, Jacques-Olivier Klein

**Affiliations:** 1 LICSEN, NIMBE, CEA, CNRS, Université Paris-Saclay, CEA Saclay 91191 Gif-sur-Yvette, France; 2 Institut d’Electronique Fondamentale, Université Paris-Sud/Paris-Saclay, CNRS, 91405 Orsay, France

## Abstract

Multiple modern applications of electronics call for inexpensive chips that can perform complex operations on natural data with limited energy. A vision for accomplishing this is implementing hardware neural networks, which fuse computation and memory, with low cost organic electronics. A challenge, however, is the implementation of synapses (analog memories) composed of such materials. In this work, we introduce robust, fastly programmable, nonvolatile organic memristive nanodevices based on electrografted redox complexes that implement synapses thanks to a wide range of accessible intermediate conductivity states. We demonstrate experimentally an elementary neural network, capable of learning functions, which combines four pairs of organic memristors as synapses and conventional electronics as neurons. Our architecture is highly resilient to issues caused by imperfect devices. It tolerates inter-device variability and an adaptable learning rule offers immunity against asymmetries in device switching. Highly compliant with conventional fabrication processes, the system can be extended to larger computing systems capable of complex cognitive tasks, as demonstrated in complementary simulations.

Biology-inspired electronics is currently attracting increasing attention as modern applications of electronics, such as biomedical systems, ubiquitous sensing, or the future Internet-of-Things, require systems able to deal with significant volumes of data, with a limited power budget. In the common von Neumann architecture of computers, an order of magnitude more energy is spent accessing memory than conducting arithmetic operations. Whilst, bio-inspired computing schemes that fuse memory and computing offer significant energy savings^1^. A fundamental bio-inspired architecture is the artificial neural network (ANN), a system where neurons are connected to each other through numerous synapses^2^. Emerging nanoscale memories known as memristive devices have been proposed as ideal hardware analogues for the latter, while the former can be realized with standard transistor devices. Therefore, a promising way to realize neuromorphic electronics is to build a hybrid system pairing transistor “neurons” interconnected via arrays of memristive devices, each which mimics a synaptic function^3^,^4^,^5^,^6^,^7^.

Memristive nanodevices can mimic synaptic weights via non-linear conductivity, controllable by applying voltage biases above characteristic device thresholds^7^,^8^. Simulated memristive ANNs have demonstrated capability to solve computational tasks using diverse algorithms^9^,^10^,^11^,^12^,^13^. Few experimental demonstrations of complete memristive ANNs exist; those built so far generally exploit inorganic devices^14^,^15^,^16^,^17^,^18^,^19^ or three terminal nanodevices^20^,^21^. However, memristive devices can also be made with organic materials that are fundamentally attractive^22^,^23^ as they offer unique advantages: low material costs, scalable fabrication via roll-to-roll imprint lithography, and compatibility with flexible substrates. These properties pave the way towards integration with embedded sensors, bio-medical devices, and other internet of things applications^24^,^25^, yet often come at the cost of slower programming relative to inorganic memristive devices or binary organic memory devices^26^,^27^. The only ANN with organic memristors uses polyaniline polymeric devices^28^, with programming durations too slow for applications (30 s per programming pulse). Here, we introduce the first demonstrator circuit capable of learning with organically-composed memristive devices as synapses that works at speeds relevant for applications (100 *μs* per programming pulse). This work exploits a unique electrochemically grafted unipolar memristive device. All weight updates are *in situ*: that is, determined by the circuit’s own learning rules^14^,^15^. We investigate how the unique properties of our organic devices impact learning efficacy both in an experimental task (emulating a linearly-separable logic gate), and simulated ones (emulating a non-linearly separable function, and classifying handwritten images).

An under-explored topic in moving from device simulations to real hardware prototypes is the intrinsic variability of memristive devices. Other imperfect behaviors, such as an asymmetric increase (SET) and decrease (RESET) of device conductance in filamentary-based memristors, resistance instability in phase change devices, and stuck-on/off effects complicate deterministic learning strategies even further^29^,^30^. In this work, we discuss and demonstrate ways to improve tolerances of generic learning rules for real-world systems. These findings suggest ways to improve not only our device, but neuromorphic supervised learning systems in general.

## Results

### Architecture

**Figure 1:**
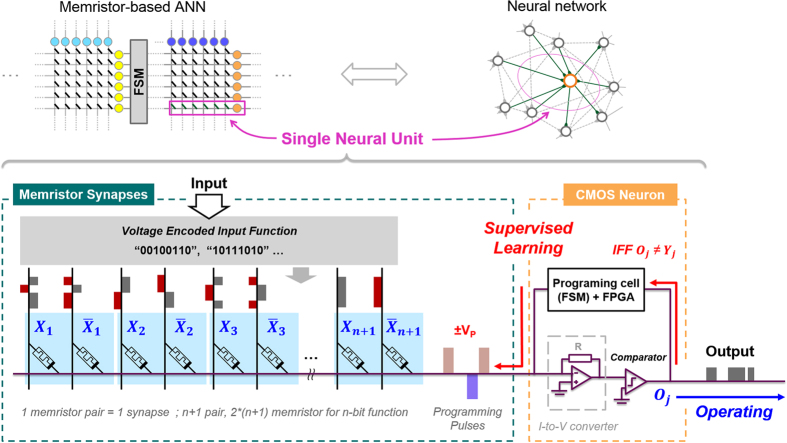
Schematic representation of the neuromorphic learning system.

The most compact architecture for organizing memristive devices in a memory structure is the passive crossbar array. However, undesired resistive paths (sneak paths) and crosstalk issues make it difficult to accurately write correct synaptic weights^31^. We obviate these concerns with a learning architecture called Neural Logic Block (NLB) resilient not only to sneak paths, but also to device variability and defects^32^,^33^. In said architecture, parallel rows of memristive devices holding trained weights (conductances) are each connected to a digital neuron unit. Each row (memristors + neuron) of the system is referred to as a Single Neuron Unit (SNU); all rows for a given layer are connected to a finite state machine (FSM) which provides programming and input learning pulses^34^. A larger ANN can be built either by cascading single neuron units (perceptrons) into multilayer perceptron architectures, or by using random or dynamic input layers (many crossbars) fed forward to a final set of parallel SNUs (single crossbar) implementing linear regression. In this work, we have experimentally realized a single SNU with 4 synapses (8 organic memristive nanodevices) that learns autonomously and in real-time. A conceptual schematic of the studied learning system is shown in Fig. 1, while its physical manifestation is detailed in the Method section (and Supplementary Fig. S1). The circuit is composed of a CMOS-based neuron programmed by a field-programmable gate array (FPGA), and a series of memristive devices mimicking synapses between differential inputs and the neuron. The circuit encodes weights using signed-synaptic pairs of memristive devices with modifiable conductivity; *n* + 1 pairs, or 2*n* + 2 memristive devices, are required to successfully map a function with *n* inputs. Each of the *n* + 1 inputs requires a negative and positive wire to separate states for that case, and negative and positive bias lines configure the entire line. In response to a set of input voltages, the sum of all conductances is proportional to current on the common post-synaptic line. After said current is converted to voltage and digitized, the sign (+, −) of the output is compared to the sign of desired function at this moment. If they are different, the programming cell applies an appropriate programming pulse to correct conductances, as defined by logic in the FPGA. Once trained for all cases of the target function, a SNU reproduces a desired signal based on its synaptic weights and input signals. This system has the ability to perfectly learn any linearly-separable function in a finite number of steps (epochs). Details on synaptic properties, learning algorithm, and CMOS neuron system are described in the following subsections.

### Synapse dynamics

Our memristive devices are metal/organic/metal junctions fabricated on oxidized silicon wafers. A series of Ti/Au electrodes with a gap of 70–100 nm are first fabricated by e-beam lithography, evaporation and lift-off. A thin organic film of covalently bounded Iron(tris-bipyridine) redox complexes (TBPFe) is then locally formed in one step by electrografting the metal electrodes in a conventional electrochemical cell. For that purpose, the three diazonium functions on the iron complexes are electrochemically reduced via cyclic voltammetry (CV) technique. The radicals formed then covalently bond the molecules to the electrodes and to each other to form a compact and robust film^35^. The redox properties of iron complexes inside the electrografted film are preserved during this process (Supplementary Fig. S4). Details on material synthesis and electro-grafting process are presented in Method section. Electrochemical deposition is fast and takes place at room temperature in a mild chemical environment. It enables the localization of the functionalization and the fine control of the film parameters, as also shown in Supplementary Fig. S3^36^,^37^, and also allows the local deposition of different compounds on a same chip. It is thus fully compliant with preexisting structures or devices on the chip, allowing heterogeneous co-integration of synapses and neurons in future designs.

**Figure 2:**
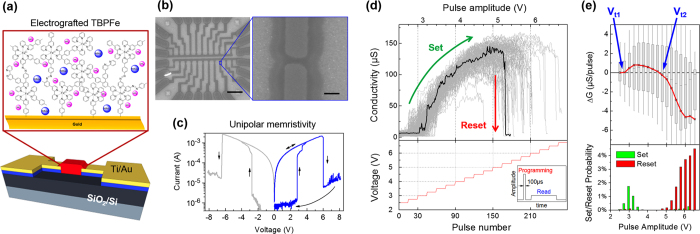
(**a**) Schematic representation of the metal/organic/metal memristor and the organic-composing active layer. (**b**) SEM image of the actual devices. Scale bar in the left and right images represent 20 *μ*m and 200 nm, respectively. (**c**) Electrical characteristics of the memristor under voltage sweeps. (**d**) Top panel: Conductivity (*G*) evolution of the device under pulses with increasing amplitude. Gray traces show all transitions and one characteristic transition (black) is highlighted. Bottom panel: amplitude of each pulse. Inset: representation of the applied waveform. (**e**) Top panel: Statistics of conductivity change (Δ*G*) versus pulse amplitudes. The gray boxes show the 25–75% probability and the whiskers are 10–90%; bottom panel: SET/RESET event (Δ*G*/*G*_0_ > 40%) probability with respect to the pulse amplitude.

A schematic representation of the device and an image of a series of organic memristive devices under scanning electron microscope (SEM) are in Fig. 2a,b. After an electric forming step, the device behaves as a unipolar conductive-filament memristor in vacuum (10^−2^ Torr). The devices possesses two thresholds: the first, at lower voltage, increases conductivity (SET); the second, at higher voltage, decreases it (RESET) as in Fig. 2c. Our device possesses *G*_Max_/*G*_Min_ ratio above 10^3^ and endurance above 2000 SET/RESET cycles. The characteristics of this device and its immunity to the scaling of surface junction imply a dynamic filamentary behavior at the active region (see Supplementary Section III-a). Although a planar structure was employed in this work, the vertical structure of the device shows comparable switching performances as horizontal ones (Supplementary Section III-b), demonstrating the possibility to be integrated in high density crossbar arrays.

To approach working conditions inside the learning system, individual devices are also characterized with impulse signals. Figure 2d shows the conductivity evolution with pulses of increasing amplitude. Series of 100 *μ*s long programming pulses are applied by increasing 0.25 V every 15 pulses. Conductance evolution is monitored at 0.5 V between each pulse. The actual applied waveform is shown in the inset of Fig. 2d. A measurement cycle begins at 2 V and ends when the device returns to a low conductance state. The black curve shows one representative cycle of the total ~2300 cycles that were applied (grey curves). These measurements show the possibility of reaching many intermediate levels with short pulses during the SET process (between 3 and 5 V), while a stable state is more difficult to obtain during RESET, which dramatically decreases conductivity above 5 V. Asymmetry between SET and RESET modes is typical of dynamic filamentary behavior, and constitutes a general limitation learning schemes must address^38^.

The top panel of Fig. 2e shows the change of conductivity (Δ*G*) as a function of pulse amplitude, based on the statistics of the former measurement. Δ*G* is defined as the difference between the device conductivity before and after applying the pulse. The red curve shows the average Δ*G* for each pulse amplitude. The two thresholds, V_t1_ and V_t2_ distinguish the SET and RESET region and are used for the learning algorithm. It should be noted that, in practice, most pulses induce very little change in memristor conductivity. The lower panel of Fig. 2e shows the SET/RESET event probability with respect to the pulse amplitude, where a SET/RESET event is counted when Δ*G*/*G*_0_ > 40%. These device characteristics are essential to optimize SET/RESET pulses amplitudes for the learning algorithm, and for the memristor to properly display synaptic function in a neuromorphic system.

### Learning algorithm

**Figure 3:**
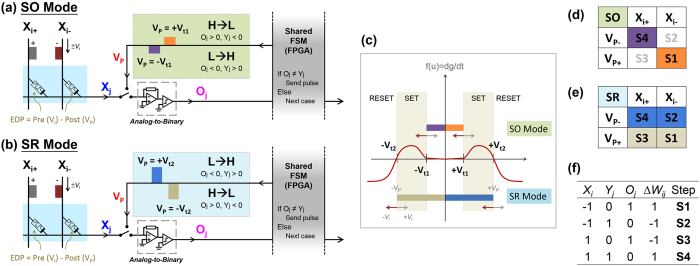
(**a**) A schematic of SO programming in an active case; while both possible correcting pulses are shown on the line, only one would be sent corresponding to depicted error case (**b**) Similar schematic showing SR programming being used to correct an error. (**c**) A diagram of device conductance evolution as it relates to appropriate thresholds for programming pulses in both modes. (**d**) Color-coded table of the active steps that SO programming implements. (**e**) Color-coded table of SR programming that implements all active steps. (**f**) Table which shows input, expected, line output, and prescribed weight change binary (sign) values at each of the four active steps.

Our system is computationally equivalent to a single-layered perceptron, a canonical classifier which, once trained, states whether a given input function belongs to a given class^39^,^40^. The Widrow-Hoff (WH) algorithm, which solves the least mean squares problem by stochastic gradient descent, provides the engine by which weights are successfully trained to map any linear function perfectly, and more complex ones imperfectly^41^,^42^. WH is implemented step-by-step; at each step (epoch), difference between expected and actual output (cost function) is computed and the appropriate adjustment is made to minimize that cost. While traditionally analog, our scheme simplifies WH into a binary form to reduce overhead. In binary WH, sign of output (*O*_*j*_) and expected (*Y*_*j*_) are compared. Therefore, there are two relevant error cases: when *O*_*j*_ < 0, *Y*_*j*_ > 0 (Low/High (LH)), and when *O*_*j*_ > 0, *Y*_*j*_ < 0 (High/Low (HL)). In the former case, WH increases the value of all pairs such that output rises to meet expected. In the latter case, WH decreases the weight of all pairs such that output falls to meet expected. Error correcting pulses *V*_*p*+_, *V*_*p*−_ sent from the finite state machine along the post-synaptic line have differential impact depending on whether the input line is high or low at that particular moment. Voltage difference across a given device (EDP) determines whether conductivity increases, decreases, or remains constant at that particular active (error-correcting) cycle of the given epoch. Since input can be high or low, two error cases become four active steps that implement WH completely: Steps 2,4 (S2,S4) improve LH error, and Steps 1,3 (S1,S3) improve HL error (Fig. 3f). A more comprehensive description of how our circuit implements binary WH is included in Supplementary Information (Section IV).

### Programming modes

Appropriate programming pulses are determined by the conductance evolution of the device^43^. Uniquely for our multi-threshold device, two thresholds offer a choice between two programming modes (Fig. 3c). As visible in Fig. 3c,d, first threshold programming uses only SET mode of the device (hereafter, Set Only (SO) mode), or two of the four active programming steps. Since the polarity of a programming pulse follows the line output *O*_*j*_, *V*_*p*+_ = *V*_*t*1+_, *V*_*p*−_ = *V*_*t*1−_ in SO mode. Two threshold programming uses both SET and RESET (hereafter, Set Reset (SR) mode), thereby implementing all four error-correcting steps with just two pulses as shown in Fig. 3e. Because conductance falls across the second threshold, and rises above the first, *V*_*p*+_ = *V*_*t*2−_, *V*_*p*−_ = *V*_*t*2+_ in SR mode. In practice, then, SO and SR modes send pulses with opposite voltage polarity to correct an equivalent error. A simplified example of each programming case is shown in Supplementary Table S1.

### Learning Results

Both SO and SR programming schemes demonstrate successful learning of diverse 3-input functions using 4 pairs of organic memristive devices (3 pairs for each of the input lines, and one pair for bias). Two characteristic learning examples (the same function attempted by both scheme) are presented here, with further successful results shown in Supplementary Section VI.

**Figure 4:**
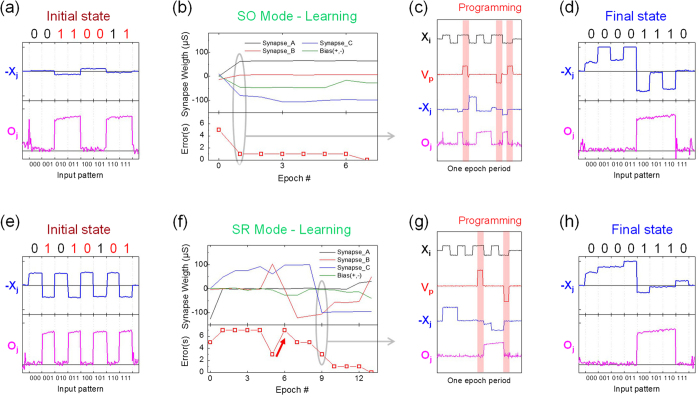
Top row: Learning of “*A* nand *B* and *C*” function (00001110) using the SO programming mode. Bottom row: Learning of the same function using the SR programming mode. (**a**,**e**) Output of I-to-V converter (blue line, -*X*_*j*_) and comparator (pink line, *O*_*j*_) showing the initial state of the system of each learning. The initial errors are marked in red. (**b**,**f**) Learning histogram showing the synaptic weights (top panel) and total errors (bottom panel) evolution at each epoch. (**c**,**g**) Example of a single learning epoch (marked with grey circle in (**b**,**f**)) showing the input *X*_*i*_ (black), programming pulses at *Y*_*j*_ (red), synaptic output -*X*_*j*_ of the I-to-V converter (blue), and digital output of the comparator *O*_*j*_ (pink) which is being compared to *Y*_*j*_. The active programming steps, when the system attempt to correct an error, are shaded red. (**d**,**h**) System output at the end of the learning, showing successful learning of the “*A* nand *B* and *C*” function.

One learning example using SO scheme is presented in Fig. 4a–d. In this case, the system is learning the “*A* nand *B* and *C*” function, i.e. a truth table output of “00001110”. To read the initial state of the SNU, a series of input signals is sequentially applied at 10 kHz rate, representing the 8 different 3-input configurations (i.e. 000, 001, …, 111). The devices can be programmed with pulses as short as 1 *μ*s, but with such short pulses, programming cannot be considered reliable. For this reason, in this demonstration, 100 *μ*s pulses are chosen. The blue line in Fig. 4a is the output of current-voltage converter (Fig. 1), which represents the total post-synaptic weight (X_*j*_) of all memristor pairs. Note that as depicted in Fig. 4 the post-synaptic value (blue line) depicted is always inverted (−X_*j*_) due to the operation of the transimpedance amplifier. The pink line shows the output of the comparator (O_*j*_), which compares actual X_*j*_ (blue) to ground (0 V). If *X*_*j*_ > 0, O_*j*_ is pulled towards “high” output (1); if *X*_*j*_ < 0 it is pulled to “low” output (0). As shown in Fig. 4a, the initial state of the SNU gives an output of “00110011” from the eight (*A*, *B*, *C*) input configurations.

Figure 4b shows the synaptic weight evolution (top panel), and error counts (bottom panel) at each epoch. Errors are gradually corrected until the system reaches an error-free state after 7 epochs. Figure 4c shows an example of every event inside one learning epoch. The black line is the input of one memristor, *X*_*i+*_, changing its sign according to the input signal at positive polarity. The blue and pink waveforms- *X*_*j*_ and *O*_*j*_ respectively- are read from the same nodes as depicted in Fig. 3a,b. The red line indicates a measurement along the wire that supplies programming pulses (*V*_*p*_ as noted on Fig. 3a,b). It shows that three pulses were applied in this particular epoch to correct the output errors for “010”, “110” and “111” inputs, respectively. It should be noted that when not in programming mode, a switch guarantees the common line is virtually grounded by the current converter, as also shown in Fig. 3a,b. Figure 4d probes the synaptic output (*X*_*j*_) and digital output (*O*_*j*_) nodes at this final state. It clearly shows that the CMOS neuron has learned the target function, “*A* nand *B* and *C*”, by producing the output “00001110” when provided a truth table as input. Figure 4e–h shows a learning example using the SR mode instead. Error counts starts at 5, and oscillates thereafter until the function is learned perfectly at epoch 13. The synaptic weights are adjusted actively during the learning process to reach the final state of “*A* nand *B* and *C*” function. Other learning examples using either SO or SR mode are presented in Supplementary Information (Supplementary Figs S7–S8). As visible, the main difference between SO and SR programming styles is that there is much less fluctuation of synaptic weight during learning in the former. This is because SO programming uses only SET, which changes a given memristive device’s conductivity more gently than RESET. It should be noted that SR programming can begin regardless of the initial state. Whilst, for SO learning, all memristive devices were first RESET before learning begins (since a physical decrease in device conductance is not accessible through this learning rule).

### Resilience to Device Imperfections

**Figure 5:**
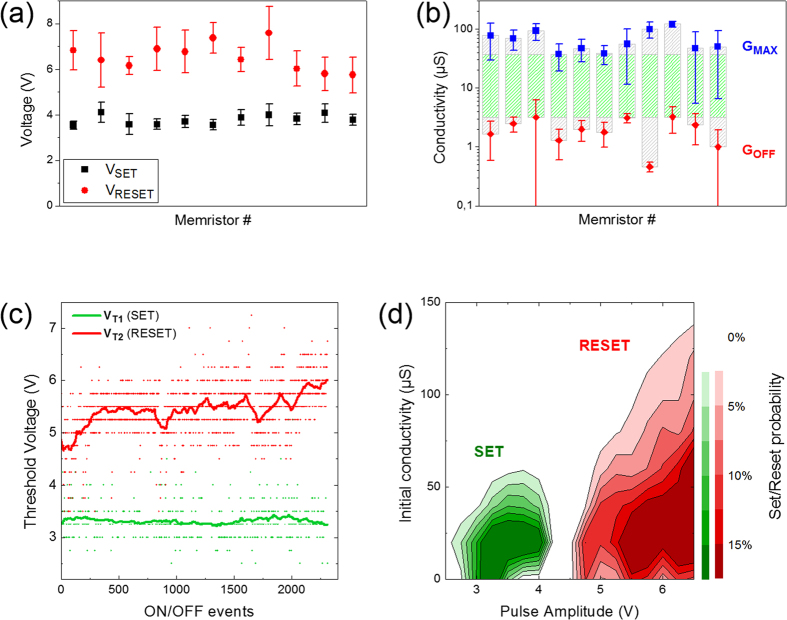
(**a**) Threshold voltages variability of 11 memristors in the same chip (*V*_t1_ in black and *V*_t2_ in red). (**b**) Maximum and minimum conductivity (*G*_Max_, *G*_Min_) variation of these 11 devices. The symbols marks the average values and the error bars indicate their standard deviations. (**c**) Evolution of SET and RESET events in numbers of On/Off cycles, extracted from the data shown in Fig. 2d. The green line shows the moving average of *V*_t1_, and the red line for the *V*_t2_. (**d**) Contour map of SET and RESET voltage with respect to their initial conductivities, *G*_0_.

Large device variability is a major setback toward the realization of robust ANNs. Figure 5a,b show the typical *V*_t1_, *V*_t2_, *G*_Max_, and *G*_Min_ variability of 11 devices (in the same row). The variation of *V*_t1_ is relatively small compared to that of *V*_t2_. This suggests the learning system should be more reliable if only *V*_t1_ is required for learning (SO mode). As for *G*_Max_ and *G*_Min_, their variations are relatively large, which is a common issue in memristive devices. Nevertheless, a relatively wide working region exists, as shown in the green zones of Fig. 5b, which permits learning in the system. The immediate effect of variability is to increase the number of epochs required to learn. For example, when using *G*_Min_ as initial states, devices with lower *G*_Min_ need extra time to correct their errors, while a lower *G*_Max_ will reduce the safe working range. The measured variability of all 11 devices on chip were 10%, 14% and 59% for V_t1_, *V*_t2_, *G*_Max_, respectively, as summarized in Supplementary Table S2. Our learning demonstration was carried out using the 8 most similar devices, reducing *G*_Max_ variability to 40%.

Although less studied, physical devices possess non-idealities beyond variable response to equivalent voltage inputs. In the case of our organic-composing device, two additional effects- asymmetric switching behavior and evolution of threshold voltages through time- have non-negligent effects on our supervised-learning system. Asymmetric behavior manifests as an imbalance between the SET/RESET processes of our device. To increase conductivity, filaments build up gradually through atomic/ion diffusion or charge transfer/trapping. By contrast, the decrease of conductivity is mostly caused by the breaking of the conductive filament, which is a violent process (Fig. 2d). This asymmetric behavior causes a fundamental problem during SR learning: instead of gradually approaching the target output (decreasing errors) at the constant weight adjustment required by WH, a dramatic RESET overshoots and in turn creates more errors than it corrects (red arrow in Fig. 4f and Supplementary Fig. S9). An immediate way to avoid this issue is to switch learning algorithm: in SO programming, memristive devices are programmed only at the first threshold, avoiding dramatic RESETs. Inversely, devices with the opposite SET/RESET asymmetry could use Reset-Only programming to avoid dramatic SET^21^. To prevent conductances from saturating (vanishing) in such a scheme, a RESET (SET) pulse would be required at every pair after a certain number of unsuccessful learning cycles.

The second physical constraint encountered with our devices is their evolution through operating time. Figure 5c shows the evolution of SET and RESET events in sequentially applied On/Off cycles. While *V*_t1_ remains constant after 2300 cycles, *V*_t2_ experiences non-negligible changes throughout the measurement period. It tends to gradually shift to higher voltage, drop to a lower voltage, and begin another upward drift cyclically. The observed behavior is compatible with repeated formation and destruction of conductive filaments. As the filament grows thicker, larger current (thus higher voltage) is required to break it. As also shown in Fig. 5d, the RESET threshold increases for a higher initial conductance. At some point, when the broken filament can no longer recover, a new thin filament is grown, which breaks at lower voltage. The aging of the device makes formation of subsequent filaments harder, and provides an explanation for why *V*_t2_ generally moves to a higher voltage over time. This impacts operation of the learning system, as the device can no longer RESET if it does not dynamically adjust the amplitude of programming pulses. While lifetime can eventually be improved by engineering, one immediate way to reduce aging is again adjustment of the learning algorithm. Switching from SR to SO programming reduces the number of On/Off events per active cycle by half (since the former implements every active step at each cycle while the latter keeps half devices in read mode at each cycle). When considering the combined effect of violent RESET and double switching activity, a switch from SR to SO could increase device and thus system lifetime substantially.

Device imperfections also affect the efficiency of SO learning, similarly increasing number of required epochs for learning. As shown in Fig. 4b (and Supplementary Fig. S7), the number of errors may remain unchanged for several epochs before decreasing again. This is due to non-linearity in the SET mode: while a programming pulse efficiently increase conductivity (Δ*G*) of the devices at lower conductance, the increment is reduced when the state prior to a pulse is already conductive. While this does not prevent learning, it does decrease efficiency. If both devices in a synaptic pair reach maximum conductivity in SO mode, i.e. are stuck at the ON state, the only solution is to RESET the system and start another learning cycle, which can complicate the function of the neuron. Moreover, if a single device is thoroughly stuck-on, learning may not be possible depending on the pair it is in and function being learned.

### Simulated learning performances

Monte-Carlo simulations of our learning system were conducted. They stress the decisiveness of device variability and conductance change asymmetry. Without variability in device thresholds and *G*_Max_, learning is always possible; the 7 three bit functions learned by our demonstrator learn in simulation every time, by mean 3.5 epochs for SO programming and mean 4.4 epochs for SR programming (when characteristic change in conductance above the first or second thresholds, Δ*G*_+_, Δ*G*_−_, respectively, are constant for every device). These results are shown in Supplementary Fig. S10. However, variability creates imperfect learning outcomes. Table 1 lists averages for 500 trials with variable nanodevices in SO mode (also see Supplementary Fig. S11a,c). As visible, the average success rate now varies slightly function-by-function. SR programming introduces the possibility of an asymmetry between the sizes of the characteristic conductance change Δ*G*_+_, Δ*G*_−_. Table 2 (also Supplementary Fig. S12a,d) shows the variable simulations for this result in a mild asymmetry case (Δ*G*_−_ is slightly larger than Δ*G*_+_).

Simulated learning results for SO, SR modes are similar: every function is learned successfully in at least four out of five cases at experimental levels of variability, while SO is slightly faster. Demonstrator learning results (Supplementary Tables S2 and S3) show that SR mode finishes faster on average (mean 12.6 epochs, SR; mean 16 epochs, SO), while requiring more programming pulses (35 SR, 26 SO). Although the sample size of successful demonstrator examples is small, characteristic device imperfections are nonetheless highlighted by this contrast. In SR mode, dramatic RESETs double the error pulses predicted by the model to be sent in one case (“A nand B or C”, Supplementary Table S4). While SR mean epoch nearly matches predicted, SO mean epoch is increased by functions that struggle against non-linear conductivity: 2 functions in particular (3NAND, “(A and B) or C”) each take 10 epochs to correct their final error case, nearly doubling epochs from 9 to 16 (Supplementary Table S3). Our simulations also highlight the fragility of SR mode; while mild intra-device asymmetry as in Table 2 is workable, stronger asymmetry (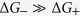) produces an average success rate of only 5–30%, depending on the function (Supplementary Fig. S12(b)). When device conductances remain ‘pinned’ to low values, insufficient weights are available to separate some cases of the truth tables of some functions from others (Supplementary Fig. S12(e)). The converse strong asymmetry case (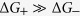) yields better results for the SR style than any other configuration (Supplementary Fig. S12(c),(f)) because it has the opposite effect of increasing the span of possible weights (conductances).

Complex, non-linearly separable functions may be perfectly learned in several layers when our system is cascaded, or imperfectly learned in one layer with larger SNUs than the one we physically realized. To show the former, we emulated a multi-layer perceptron system by feeding forward functions learned in a first layer to build the truth tables of linearly non-separable functions in subsequent layers^40^. Similarly to^44^, which showed a multi-layer memristive system can learn AND and NOT in a first layer with two SNUs and subsequently the 2-bit XOR function with another in the second, we learned the 3-bit XOR function (01101001). Three SNUs in the first layer and one SNU in the second (32 organic memristive devices total) are required to resolve this problem. The function is perfectly learned with perfect devices; when approximating experimental levels of inter-device variability, both programming styles can still successfully resolve the problem: mean 79% success rate is obtained for SR mode (Supplementary Fig. S13(a),(c)) and 71% for SO mode (Supplementary Fig. S13(b),(d)).

To demonstrate the latter, a canonical image recognition task (the MNIST database of handwritten digits^45^) was attempted using a simulated crossbar composed of 15,680 of our organic memristive synapses. Ten separate perceptrons or SNUs- one for each digit class- each require double the number of synaptic devices as pixels (*p* = 784) to encode positive and negative weights. All see a given example digit simultaneously, and all correct weights simultaneously as given by the error case (if there is one). By the end of training, the Widrow-Hoff algorithm has progressively adjusted weights (device conductances) such that each SNU emulates a binary classifier between the chosen class and all others (geometrically, this solution is a hyperplane in *p*-dimensional space). As visible in Supplementary Fig. S14, performance on this task is robust: top performances for SO and SR modes are at 86% and 88% respectively, and mild dispersions around the thresholds and *G*_max_ are not noticeably detrimental. While SR mode continues to slightly improve performance as it receives more training samples, SO performance markedly declines after a certain number are given due to a saturation of all devices towards *G*_max_. Similarly to the logic gate case, SR is superior in the symmetric case but substantially degrades when asymmetry (violent RESETs) are considered. Figure S14 shows that in the worst case- every RESET pulse is double the power of SET- both variable and uniform devices fail to consistently resolve the MNIST task at greater then 70% accuracy even when many samples are given. Supplementary Fig. S15 further demonstrates that even simulated systems with worse than experimental levels of variability continue to perform well on the task. SR is more resilient than SO as dispersions increase, which may relate to devices with very low *G*_max_ becoming ‘stuck-on’ early (whereas they could be decreased in SR). The task shows that a large memristive perceptron using binary Widrow-Hoff approaches the natural accuracy limit of a perfect perceptron implemented in software (90%). The higher performance on MNIST than the 3-XOR problem highlights a trade-off between exactness and efficiency in hardware ANNs. With logic functions every case of the truth table must be perfectly emulated, while in classification problems aggregate dynamics allows for approximately correct answers to emerge.

## Discussion

While our system is not the first to demonstrate a perceptron built with organic memristive synapses^28^, our memristive learning system is the first to scale to speeds relevant to applications in real-time data processing, to co-integrate hardware neurons with memristive synapses in a fully embedded learning system, and to analyze and demonstrate robust learning over characteristic dispersions in device behavior. These advances significantly improve the prospects for ANNs built from organic nanosynpases.

The existing demonstrator relied upon a pulse generator and a FPGA exterior to the same chip that contains the nanodevices, yet a fully on-chip implementation of the neural network can be achieved by associating organic transistors directly with the nanosynapses. Additionally, the organic devices could themselves be leveraged in the finite state machine implementation, as recently demonstrated^46^. In this scheme, each SNU also includes a pair of memristive devices that uses a concept known as stateful logic to signal the correct configuration while additional transistors connect programming or common lines at appropriate moments. This circuitry suffices to route error-correcting pulses to SNUs with the appropriate error case. While an on-board finite state machine comes with an area overhead to the crossbar, one such system may be shared by several interconnecting crossbar arrays (layers). Additionally, our system may be integrated with low cost, organic sensors^47^. Associating sensing to a hardware neural network could allow a particularity cost and energy effective “smart sensor” feature, essential for many biomedical and ubiquitous sensing applications.

Our proposed architecture allows for perfect learning of linear functions and imperfect learning of non-linear functions; while the experimental learning of logic gates demonstrates the former, our simulated results demonstrate the latter. Both approaches show resilience to device imperfections, which makes them especially attractive to neuromorphic designers. As demonstrated, SNUs can accept inputs more complex than a truth table and be chained together. This suggests that systems with many layers (crossbars), each composed of several SNUs, can be built, as pictured in Fig. 1. Such systems may be able to solve canonical tasks with greater accuracy then we showed here, as well as to cope with real-time classification problems from sparse inputs, such as sensor data. Memristive implementations of the back-propagation algorithm, e.g. the Manhattan Rule^48^,^49^ have achieved promising simulated results. Another approach trains multilayer memristive systems on more difficult tasks using the concurrent learning algorithm^50^,^51^. Since all SNUs in an array can be corrected with only two programming pulses per epoch, binary Widrow-Hoff with conditional error-correction may be more energy efficient than both as well as ref. 28. As our algorithm is also a variant of stochastic gradient descent, additional circuitry would be needed to store error layer-by-layer. An alternate solution that avoids overhead entirely needs not train the weights of devices in first layers, but rather exploits their variability or time-dynamics as a projection space which is thereafter regressed. This approach has been shown to integrate well with filamentary memristive nanodevices, and may offer a flexible solution space between static (feed forward) and time-delayed (reservoir or liquid state) solutions as well^52^,^53^,^54^. Our MNIST study shows that a linear regression filter can be implemented on-chip in the last layer (crossbar) by a number of SNUs corresponding to the number of outputs (classes).

In summary, we have successfully demonstrated the supervised learning capability of a small neuromorphic learning system that integrates a novel organic memristive device as synapse. The complex conductance evolution of this device offers flexibility in programming voltage and corresponding learning style; both SET and RESET regimes can be exploited to effect suggested weight changes (second threshold/SR learning), or only SET (first threshold/SO learning). Examples of the learning of 3-input logic functions are shown for both styles and are compared to simulations. Learning is also possible on more complex tasks than the one attempted with our demonstrator. In all these cases the system shows high tolerance to inter-device variability in both first and second threshold styles, which speaks to the power of our supervised learning algorithm. However, there is less resilience to imperfections involving the RESET mode and asymmetry between characteristic SET and RESET conductance characteristic changes. For this reason, while theoretically less efficient, first threshold (SO) programming remains a better choice for neuromorphic systems built with our present devices. Based on our insights, abundant opportunities exist to further improve our learning algorithm and device to enhance reliability. In particular, device engineering which improves RESET performance could yield even more efficient pulse programming and thus considerable energy savings in larger systems, while a more adaptive algorithm may enhance flexibility to characteristic imperfections that are harder to eliminate (such as device aging). Our physically realized learning system is the primitive for a computationally universal system, for which works are currently ongoing. Ultimately, our work aims towards the realization of fully on-chip, organic-based supervised learning systems with low production cost, which operate with the analog computing power necessary to tackle unconventional problems in unconventional electronics environments.

## Methods

### Material synthesis

The iron(II) *tris*–bipyridine complex with diazonium functions was synthesized according to the procedure depicted in Supplementary Fig. S2. All reagents and chemicals were purchased from Aldrich and used as received. 4′-(4-Aminophenyl)-2,2′-bipyridine (**BipyNH**_**2**_) was prepared according to a procedure described previously^55^. Characterization techniques: NMR spectra were recorded with a Bruker ADVANCE DRX 400 (400 MHz). Chemical shifts *δ* are expressed in ppm relative to tetra-methylsilane (TMS). Infrared spectroscopy (IR) was realized with a Bruker Vertex 70 spectrometer (resolution 2 cm^−1^, 24 scans collected, MCT detector) equipped with a Pike Miracle plate for ATR. UV-Vis spectra were recorded with a Perkin Elmer Lambda 650 spectrometer. Mass spectra were acquired in the positive mode on a LCQ-ion trap Thermofinnigan spectrometer equipped with an electrospray source (MS-ESI).

(a) [Fe(Bipy-ph-NH_2_)_3_][PF_6_^−^]_2_ (**FeNH**_**2**_). A solution of iron(II) tetrafluoroborate hexahydrate (91 mg, 0.33 eq.) and **BipyNH**_**2**_ (200 mg, 0.81 mmol) in ethylene glycol (4 mL) was heated at 60 °C for 5 min. Afterward, 100 mL of water saturated KPF_6_ were added. The precipitate obtained was filtrated and washed several times with diethyl ether to give **FeNH**_**2**_ as a purple solid (270 mg; 92% yield). ^1^H NMR (400 MHz, DMSO_−6_, *δ*): 9.12 (d, J = 7.9 Hz, 1H), 9.01 (s, 1H), 8.23 (t, J = 7.2 Hz, 1H), 7.83 (d, J = 8.0 Hz, 2H), 7.76 (m, 1H), 7.60–7.45 (m, 2H), 7.28 (d, J = 6.1 Hz, 0.5H), 7.17 (d, J = 6.1 Hz, 0.5H), 6.69 (d, J = 8.0 Hz, 2H), 5.87 (s, 2H). ^13^C NMR (50.32 MHz, DMSO-d_6_, *δ*): 159.3, 158.6, 151.7, 149.0, 138.2, 130.6, 128.8, 128.3 (2C), 127.3, 124.0, 122.1, 120.5, 118.8, 113.8 (2C). IR *ν* = 3367, 3098, 1594, 1524, 1470, 1437, 1411, 1330, 1261, 1189, 1054, 826, 787 cm^−1^. UV-vis (*acetonitrile*): *λ*_*max*_ = 545, 508 (sh), 369 nm. MS (ESI) m/z: calcd for C4_8_H_3__9_FeN_9_^2+^, 853.20; found, 398.8 (M - 2PF_6_^−^).

(b) [Fe(Bipy-ph-N_2_^+^)_3_][PF_6_^−^ or BF_4_^−^]_5_ (**FeN**_**2**_^**+**^). Under argon, nitrosium tetrafluoroborate salt (13 mg, 1.2 eq.) was added directly to a degassed solution at −40 °C of **FeNH**_**2**_ (100 mg, 0.1 mmol) dissolved in dry acetonitrile (5 mL). After 5 min of stirring at this temperature, diethyl ether was added until a precipitate came out. The precipitate was filtrated, washed several times with diethyl ether to give a purple powder **FeN**_**2**_^**+**^ (125 mg, quantitative yield). ^1^H NMR (400 MHz, CD_3_CN, *δ*): 8.88 (d, *J* = 4.2 Hz, 1H), 8.75 (dd, *J* = 7.5 Hz, *J* = 4.5 Hz, 1H), 8.67 (d, *J* = 8.7 Hz, 2H), 8.34 (d, *J* = 8.7 Hz, 2H), 8.21 (t, *J* = 7.5 Hz, 1H), 7.80–7.40 (m, 4H). IR *ν* = 3107, 2280 (N ≡ N), 1583, 1540, 1468, 1438, 1402, 1333, 1285, 1233, 1022, 826, 785, 750 cm^−1^.

### Electrografting of Iron complex FeN_2_^+^

The electrochemical grafting was conducted in a single-compartment three-electrode cell with a potentiostat (Model VSP Bio-Logic SAS) in a glovebox. Ag/AgNO_3_ (10 mM) electrode and a platinum wire served as reference and counter electrode, respectively. All potentials in the following are referenced to Ag/AgNO_3_. The silicon substrate with the patterned gold working electrodes was completely immersed in a solution of **FeN**_**2**_^**+**^ (34 mg/L) dissolved in tetrabutylammonium hexafluorophosphate (0.1 M)/acetonitrile electrolyte. The gold electrodes were connected with a passivated tungsten tip. Chrono-potentiometry technique (5 s at −8 *μ*A) was used to make smooth thin films of covalently bounded Iron(tris-bipyridine) (**TBPFe**) complexes (See Supplementary Fig. S3), AFM image and height profiles of modified electrodes. Cyclic voltametry (CV) technique was used to grow thicker film for the memristive device.

The electrochemical properties of an electrodeposited 14 nm-thick film were studied by CV in a medium of analysis free from the pristine complex (Supplementary Fig. S4). CV shows the characteristic peaks of the complexes grafted on the electrode, i.e. a reversible wave in oxidation (0.78 V vs Ag/Ag^+^), two reversible waves in reduction (−1.58 V and −1.76 V). It is worth to note that the potentials of the metal complexes inside the polymer films were closed to those for dissolved iron trisbipyridines complexes.

### Experimental details

The previously described electrografted memristive synapses have been integrated in a chip with 22 total devices (11 on two individual lines), where they can be accessed individually and collectively on each of their ports using individual input lines along with the common line. Once put in vacuum using an accessory pump (Alcatel ACP 286), said chip is connected to a custom-designed printed circuit board (PCB) connected to a power source (Agilent E3631A). Within the PCB, devices are connected to accessory circuitry such as the comparator, current to voltage converter, etc needed to read line output. In addition, the PCB contains components for electrostatic discharge (ESD) protection. The board is directly connected to an FGPA (Altera Cyclone DE2-70), which both reads from the devices and sends appropriate programming pulses by using the onboard logic (FSM) with which it has been programmed by accompanying generic Altera software (Quartus). A custom NIOS softcore dedicated to interface with a PC and corresponding graphical user interface were coded to allow for real time user control of the functions that have been loaded onto the FPGA during programming. Functions such as erase, read, and learning modes (single epoch, or continuous) can be applied to devices subsequently. An oscilloscope (Agilent MSO 6014A) probes key electrical ports and is also connected to the PC for real time data collection assisted by LabView. A representation of the actual setup is available in Supplementary Fig. S1.

### Simulation details

Monte-Carlo (MC) simulations for both single SNU (experimental) and multilayer SNU (XOR) tasks were conducted using a functional model of the device and learning system written in Matlab. For each function being learned, 500 MC iterations- each which begins with a random set of low initial conductances among 8 simulated memristive devices- are simulated. When variability mode is enacted in the SO case, at each iteration, inter-device variability is additionally emulated by picking random first threshold and *G*_Max_ values for each memristive device from a normal distribution around a characteristic value with *σ*(*G*_Max_) = 40%, *σ*(*V*_t1_) = 10% and Δ*G*_+_ pegged as 10%*G*_Max_. Since each device has a different *G*_Max_ value, each also evolves conductance differently. For SR simulations including inter-device variability, *σ*(*V*_t1_) = *σ*(*V*_t2_) = 10%, and Δ*G*_+_ = 15%*G*_Max_ Δ*G*_−_ = 20%*G*_Max_ for each respective memristive device (this case assumes the powerful second threshold decreases we observed experimentally and a relatively powerful first threshold increase). Every simulated system in a given iteration is granted 50 epochs; if it does not solve all cases of the target function’s truth table by the final epoch using WH to adjust weights, learning is considered a failure. Average success rate is given by the number of successful trials divided by iterations. Errors corrected and epoch completed are obtained by taking mean values over all iterations (if the function failed to learn, epoch learned in that case is counted as the maximum (50)).

Simulation in the MNIST case was achieved by presenting images from the MNIST database as a set of positive/negative voltage spikes to the input of a crossbar with 2 * *p* input wires and 10 output wires during many moments in separated testing and training phases. In the training phase the output of the wires immediately signals which correcting pulses are to be sent to each row, and weights are adjusted in a moment after but before the next sample is sent. The adjustments are precisely the on-chip Widrow-Hoff algorithm as described in Section IV with the following modification: an input index *i* with a pixel (spike) applied are given as X_*i*+_ = +1.5 *V* and X_*i*−_ = −1.5 *V*, and no spike at that iteration index receives the opposite (X_*i*+_ = −1.5 *V* and X_*i*−_ = 1.5). During testing phase, digits are presented at read voltage so as not to disturb weight matrix *W*. Guesses are determined via dot product of presented images as voltage spikes and weight matrix *W*, which outputs each row of crossbar as currents; row *j* with max current is guess. Classification percentage is computed as the number of correct guesses made divided by the quantity of the entire MNIST test set (10,000). 15,680 organic memristive nanodevices were simulated either uniformly or at dispersion parameters noted in the captions/axes of Figs S14 and 15 in order to achieve the stated results. Characteristic conductance change was either 5%*G*_Max_ or 2.5%*G*_Max_, as noted in captions of Figs S14 and 15. Both reducing programming pulse length and reducing voltage (EDP) to less than the experimental values could allow us to access such smaller changes.

## Additional Information

**How to cite this article**: Lin, Y.-P. *et al*. Physical Realization of a Supervised Learning System Built with Organic Memristive Synapses. *Sci. Rep.* **6**, 31932; doi: 10.1038/srep31932 (2016).

## Supplementary Material

Supplementary Information

## Figures and Tables

**Table 1:** Simulated results for First Threshold (SO) Learning assuming experimental variability parameters

	Avg Success	Avg Epoch	Avg Errors
3NAND	87%	9.36	25.33
(A and B) or C	80%	12.77	36.73
A → (B → C)	88%	9.05	30.02
A nand B or C	86%	9.98	28.1
MAJ	91%	8.54	25.08
A and (B or C)	83%	11.35	32.76
3AND	85%	10.73	30.1

**Table 2:** Simulated results for Second Threshold (SR) Learning assuming experimental variability parameters.

	Avg Success	Avg Epoch	Avg Errors
3NAND	83%	13.44	26.34
(A and B) or C	80%	14.81	25.11
A → (B → C)	88%	11.34	13.11
A nand B or C	82%	14.04	26.04
MAJ	84%	13.07	19.01
A and (B or C)	85%	12.94	22.95
3AND	82%	14.85	26.71
