# Supplementary material for: Physical Realization of a Supervised Learning System Built with Organic Memristive Synapses
Source: Sci Rep. 2016 Sep 7;6:31932. doi: 10.1038/srep31932 (PMC5013285; doi:10.1038/srep31932)
Supplement: Supplementary Information [file srep31932-s1.pdf]

Supplementary information for *"Physical Realization of a Supervised Learning System Built with Organic Memristive Synapses"*

Yu-Pu Lin[1,+], Christopher H. Bennett[2,+], Théo Cabaret[1], Damir Vodenicarevic[2], Djaafar Chabi[2], Damien Querlioz[2], Bruno Jousselme[1], Vincent Derycke[1], and Jacques-Olivier Klein[2]

[1]LICSEN, NIMBE, CEA, CNRS, Université Paris-Saclay, CEA Saclay 91191 Gif-sur-Yvette, France.
[2]Institut d'Electronique Fondamentale, Université Paris-Sud/Paris-Saclay, CNRS, 91405 Orsay, France.
[+]The authors contributed equally to this work.

I Experimental set up

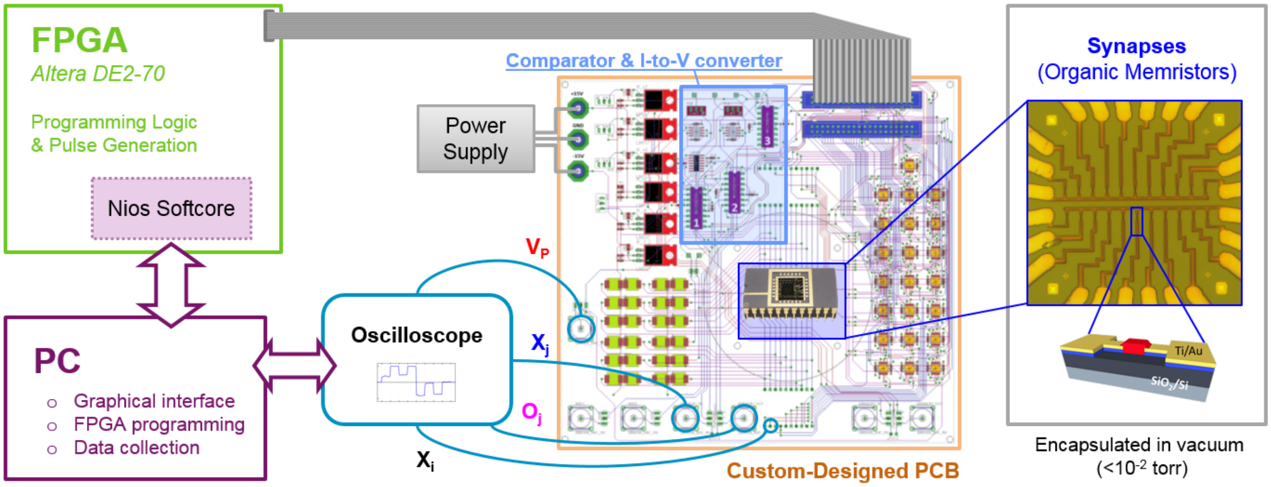

Figure S1. Organic memristive nanodevices, placed within a custom designed PCB board connected to a power source, are put into vacuum using an accessory pump. Within the PCB board, the devices are connected to accessory circuitry (comparator, current to voltage converter), needed to interpret line output. The PCB board contains additional components for electrostatic discharge (ESD) protection. The board is connected to an FGPA, which sends appropriate programming pulses using the logic (FSM) it was programmed with before the learning process began using a PC. A NIOS softcore was created to allow for real time user control of the functions that have been loaded onto the FPGA during programming via the connected PC interface. Functions such as erase, read, and learning modes (single epoch, or continuous) can be applied to devices subsequently. Finally, an oscilloscope probes the key electrical ports noted in Figure 3,4: line output X_j, comparator output O_j, programming pulses V_p, and expected function Y_j. The oscilloscope is also connected to the PC for real time data collection.

II Supporting figures for experimental methods

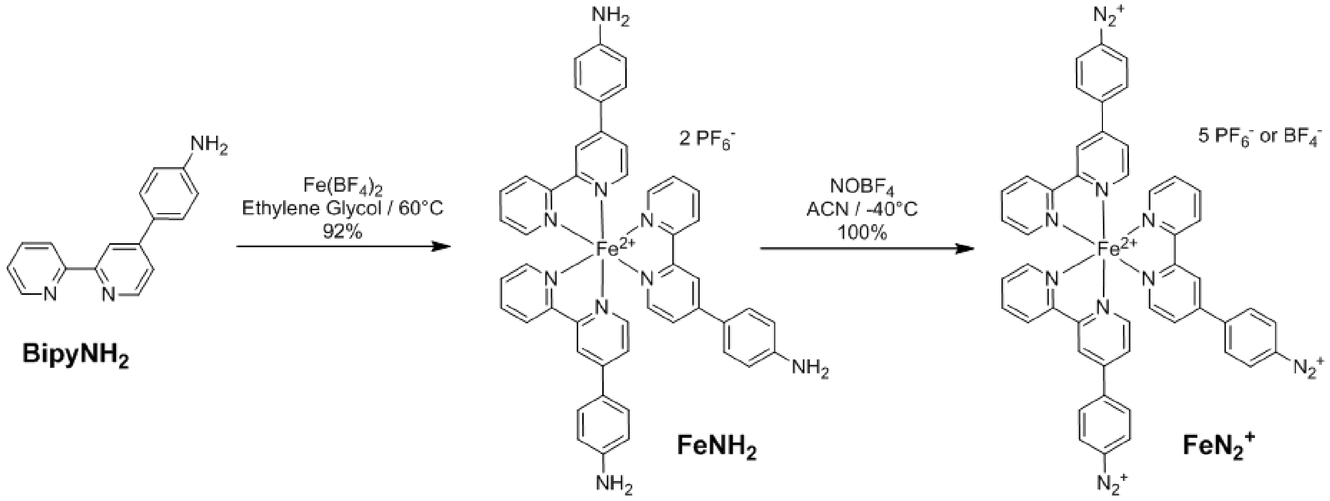

Figure S2. Synthesis pathway of iron complex bearing three diazonium-salts functions.

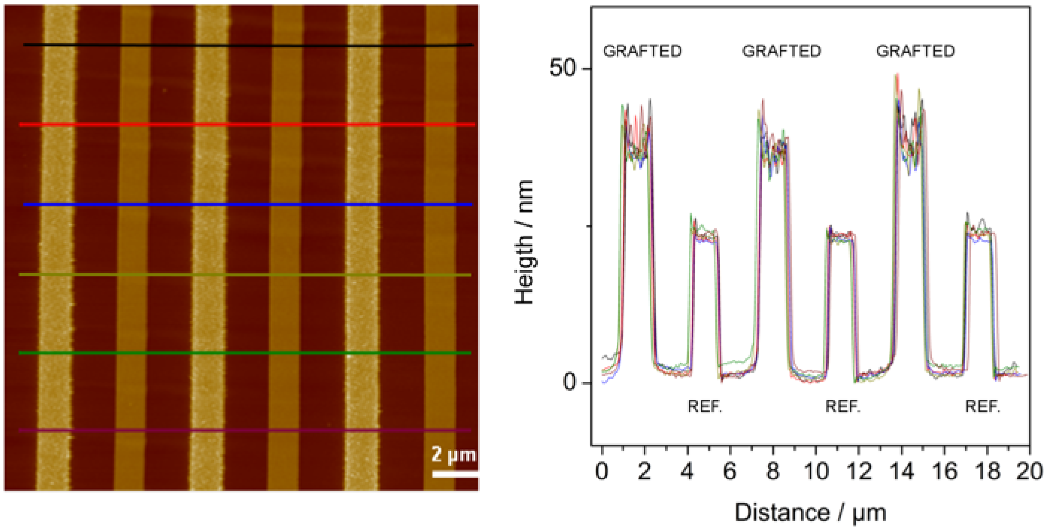

Figure S3. AFM image of electrodes electro-functionalized with $\mathbf{FeN_2}^+$ (2×10^{-5} mol·L^{-1}) in 0.1M NBu_4PF_6/acetonitrile electrolyte using chrono-potentiometry technique (5s at -8μA). Electrodes labeled *REF*. were not grafted and serve as reference. The AFM height profiles show the homogeneity of the films thickness along each electrode and between electrodes grafted separately.

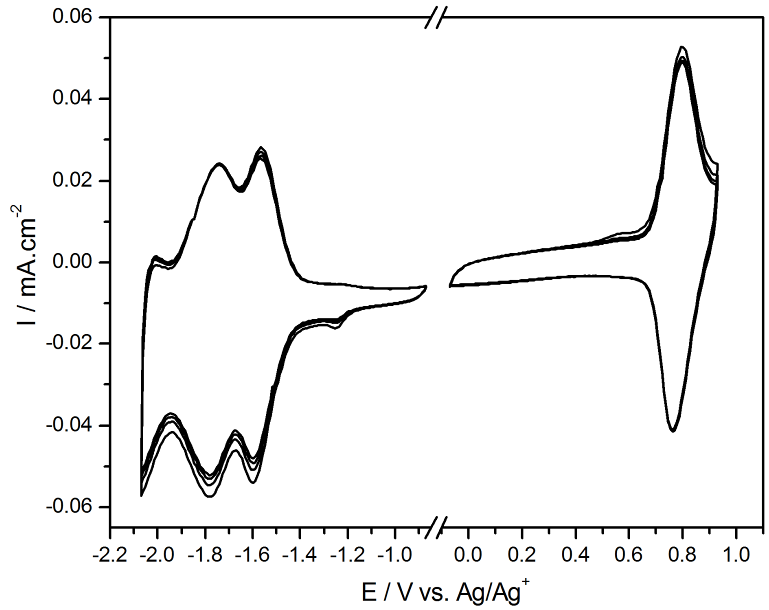

Figure S4. CV of a gold electrode modified by $\mathbf{FeN_2^+}$ in 0.1 M Bu_4NPF_6/acetonitrile, 100 $mV{\cdot}s^{-1}$.

III Supporting information for the memristive device

a Discussion on device operating mechanism

The operating mechanisms of organic memristors are usually difficult to fully identify and there is little agreement in the literature on the filamentary vs. bulk nature of the switching and on the respective impact of the different elements (electrodes, organics, and substrates). In our case, the unipolar filamentary nature of the switching is clearly established based notably on the two following arguments:

- Firstly, the device properties (threshold voltages and the maximum conductivity) do not scale with the junction area. As displayed in figure S5, devices with different junction area spanning a very large range display very similar characteristics. This implies that the change of conductivity only affects a small area rather than the entire junction.

- Secondly, as shown in figure 5d (main text), the RESET threshold varies with the initial conductivity, while the SET threshold does not. The higher the initial conductivity, the higher the voltage required to RESET the device. This can be explained if the organic memristor varies its conductivity through the formation and rupture of conductive filaments. It is indeed expected that when a larger conductive filament is formed, a higher energy is needed to break it.

Concerning the nature of the filaments, we excluded the role of the metal electrodes by changing the nature of one or both metals (including by using carbon nanotubes as electrodes). We also excluded the role of the surface (in planar junctions) notably by studying organic memristors on flexible organic substrates. Electrochemical characterizations show clear reversible memory effects in solution associated with redox process. Yet, in a device configuration, we cannot presently fully exclude the formation of carbon-based filaments originating from a degradation of the redox film during the electrical forming step. These elements will be presented separately in a publication specifically dedicated to the operating mechanism of electrografted organic memristors.

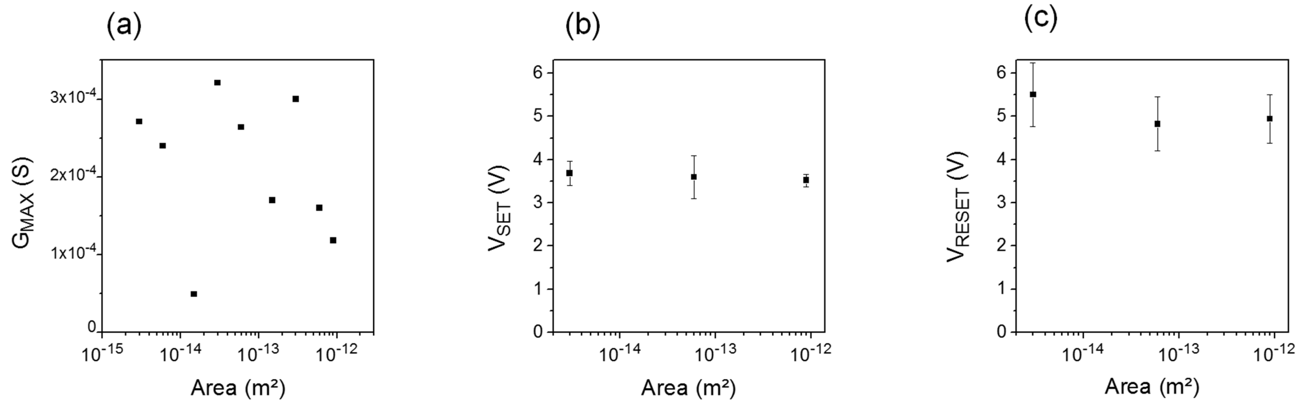

Figure S5. Dependency of (a) the maximum conductivity (G_{MAX}), (b) SET threshold and (c) RESET threshold to the cross section area of the channel.

b Vertical device structures

The vertical memristive junctions are fabricated by electrografting a thin organic film (of typically 20-30 nm) on bottom gold electrodes and then by direct deposition of a second gold electrode. Both bottom and top electrodes are fabricated by e-beam lithography, evaporation and lift-off. A schematic representation and a SEM image of such vertical device are shown in figure S6a,b. After electrical forming, the vertical device exhibit comparable characteristics to the planar ones (figure S6c): similar SET (2.5-3V) and RESET (3.7-4V) bias ranges and a wide range of accessible intermediate conductivity states. The endurance of such vertical configuration is lower than for planar structures and the device failure is characterized by devices getting shorten. This is most probably due to partial deterioration of the organic layer upon metal evaporation. Work is underway on the electrografting parameters to improve the compactness of the film to limit such degradation.

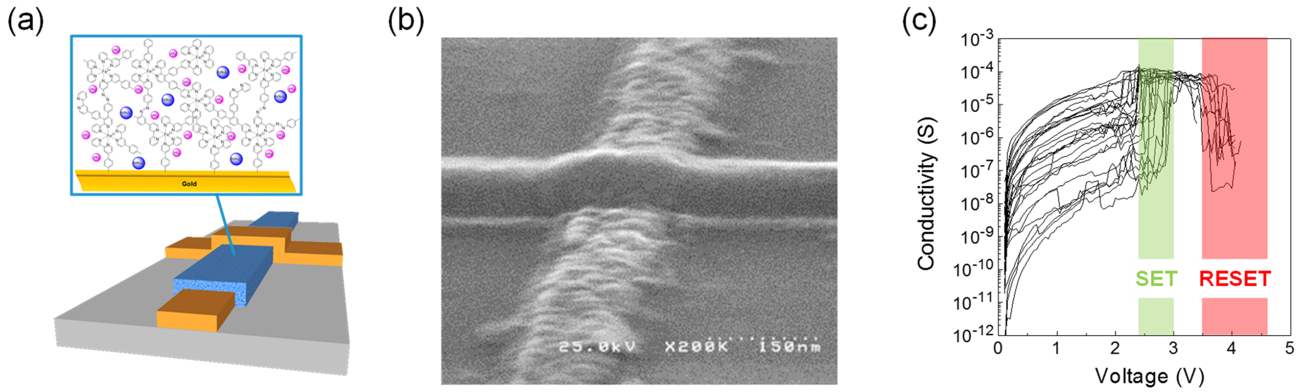

Figure S6. (a) Schematic representation and (b) SEM image of a vertical memristor. (c) Representative IV characteristics of the vertical devices upon application of a series of voltage sweeps with different final states. The conductivity at a read bias of 1V shows the accessibility of different intermediate states.

IV Detail on Learning Algorithm

A binary version of WH is implemented to train the weight of a memristive device pair W_i such that it maps input X_i to output Y_j. Weight change (Δ) refers to a fixed change ($\alpha = 1$) implemented at that case of the function in order to reduce error (difference between expected Y_j and the actual line output V_j):

$$\Delta W_i = \alpha X_i (Y_j - V_j) \tag{1}$$

Using this rule, $n+1$ memristive pairs or $2n+2$ memristive devices are required to segregate n-input functions; each of the $n+1$ inputs requires a negative and positive wire to separate states for that case, and negative and positive bias lines configure the entire line. Explicitly, a given input X_i divides into two sign-symmetric input wires (X_{i+}, X_{i-}) that serve as input to two organic memristive devices M_{i+}, M_{i-}, with conductances G_{i+}, G_{i-}, respectively. A pair i produces synaptic weight W_i as follows:

$$W_i = K(G_{i+} - G_{i-}) \tag{2}$$

where K is a normalizing factor. In response to a set of inputs $X_1 \cdots X_{n+1}$, a shared post-synaptic potential X_j is obtained automatically by linear combination of pair weights:

$$X_j = \sum_{i=1}^{n+1} = W_i X i \tag{3}$$

X_j is converted from current to voltage and subsequently from an analog to a digital value ($sign(V_j)$) after it passes through a comparator set to ground. This has the effect of inverting the sign of X_j. For every (2^n, where n is the number of bits) case of the function's truth table, a common FSM checks whether the SNU's post-comparator output (O_j) is the same sign as expected (Y_j). If so, the next case is checked; else, if $sign(Y_j) \neq sign(O_j)$, a programming pulse is sent.

	Error case	Actually applied voltages (V)					Individual conductance (G) and Pair Weights Changes			Step(s)
		X_{i+}	X_{i-}	V_P	EDP+	EDP-	ΔG_+	ΔG_-	ΔW_{ij}	
SO Mode	1 (H→L)	+1.5	-1.5	+3*	-1.5	-4.5	-	↗	-1 (↘)	S1
	1 (L→H)	+1.5	-1.5	-3	4.5	1.5	↗	-	1 (↗)	S4
SR Mode	1 (H→L)	+1.5	-1.5	-5*	6.5	4.5	↘	↗	-1 (↘)	S3,S1
	1 (L→H)	+1.5	-1.5	+5	-4.5	-6.5	↗	↘	1 (↗)	S4,S2

*Correspond to V_{P+} in Fig. 3d,e of the main text. (The unmarked ones correspond to V_{P-})

Table S1. Simplified example of each programming case as presented in Fig. 3 of the main text, assuming V_t1=3V, V_t2=5V, V_i=1.5V, and electrical differential potential across the memristor (EDP) = $V_i - V_P$. X_{i+} and X_{i-} stand for the input of one memristor pair, S1-S4 are active step to correct errors according to the WH table, as also shown in Fig. 3 of the main text.

V Device performances variability

	Mean	Standard deviation	Variability
V_{t1} (V)	3.78	0.39	10%
V_{t2} (V)	6.72	0.97	14%
G_{Max} (μS)	69.5	41.2	59%
*G_{Max} (μS)	73.3	29.0	40%

*G_{Max} of the 8 memristors used in learning system prototype.

Table S2. Typical variability of memristive performances extracted from 11 devices of the same chip.

VI Complete Demonstrator Learning Performance

7 linearly-separable 3-bit functions were attempted by our demonstrator using both programming styles (SO,SR). Of the 7 functions attempted, 5 were successfully learned in both cases. Success cases are highlighted below for both modes. Although a very small sample, mean values show that SR completes faster but 'wastes' many pulses in the process. Conversely, SO takes more epochs but less pulses. The former is explained by RESET overshoots; the latter, by 'sticky' devices that take many epochs to reach a high enough conductance.

	Epoch Learned	Errors Corrected
3NAND	33	38
(A and B) or C	21	31
A $\rightarrow$ (B $\rightarrow$ C)	9	26
A nand B or C	7	10
MAJ	12	24
**Mean**	**16.2**	**25.8**

Table S3. All Functions learned Successfully by demonstrator using First Threshold (SO) Programming

	Epoch Learned	Errors Corrected
MIN	14	39
A nand B or C	13	64
MAJ	6	13
A and (B or C)	8	20
3AND	22	39
**Mean**	**12.6**	**35**

Table S4. All Functions learned Successfully by demonstrator using Second Threshold (SR) Programming

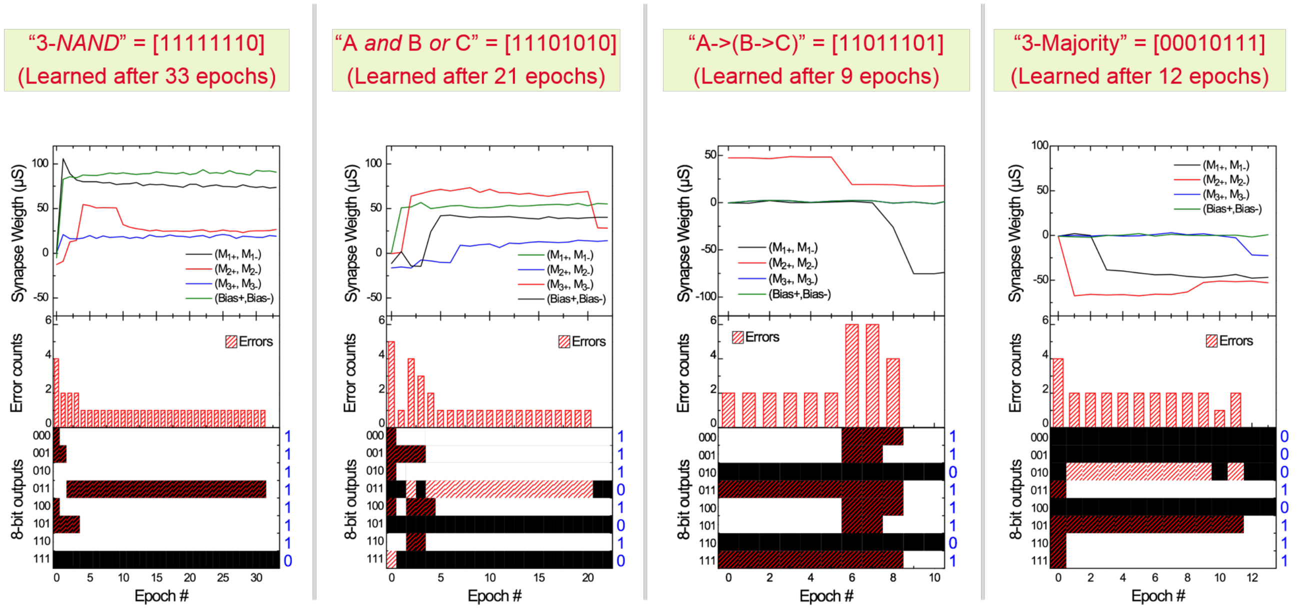

Figure S7. Examples of learning 3-input logic function using SO Mode. For each example, the top panel shows the evolution of synaptic weight; the middle panel shows the error counts after each epoch; the bottm panel shows the digital output of each case after each epoch, where error(s) are marked in red. As shown in the middle panels, the system often get stuck at an intermediate state with one or two errors due to the "nonlinear SET" behavior of the memristor.

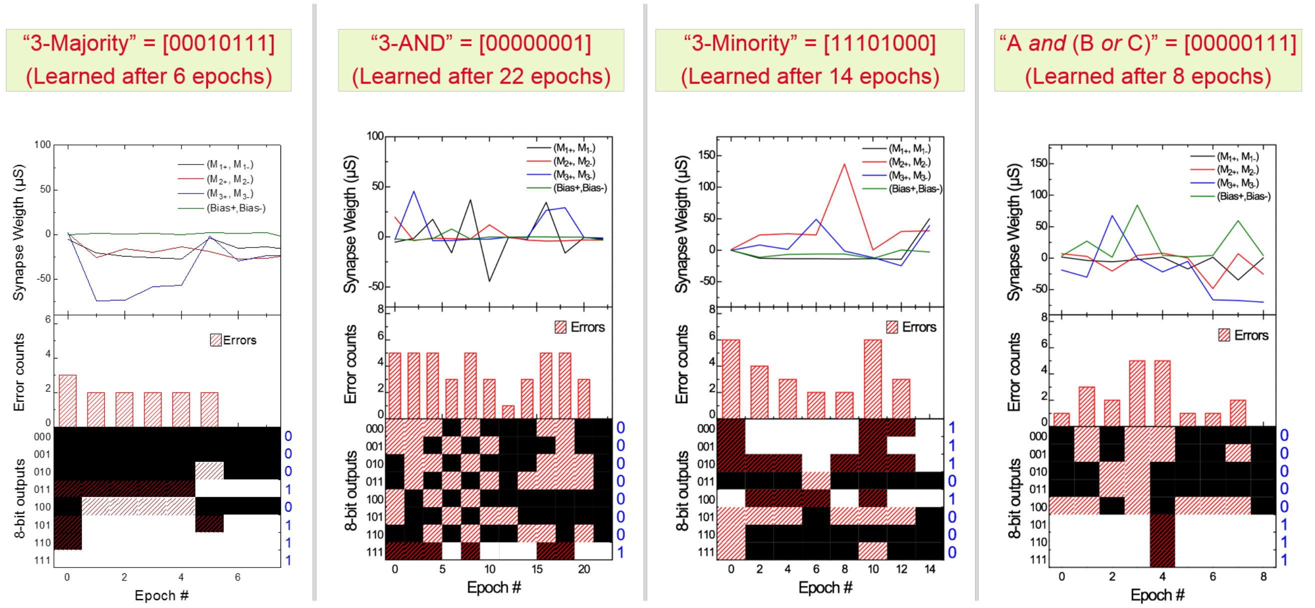

Figure S8. Examples of learning 3-input logic function using SR Mode. For each example, the top panel shows the evolution of synaptic weight; the middle panel shows the error counts after each epoch; the bottm panel shows the digital output of each case after each epoch, where error(s) are marked in red. As shown in the top panels, there is more fluctuation of the synaptic weights when using Mode 2.

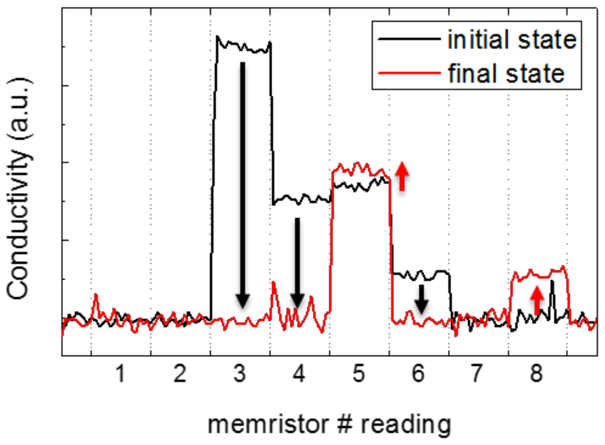

Figure S9. Conductivity of the 8 memristors before and after the learning epoch indicated by the red arrow in figure 4f of the main text. It shows that RESET processes are dramatic in this case. It decrease the device conductivity to it minimum, while SET processes increase conductivity more gently.

VII Simulated Learning Performance

a Experimental Task

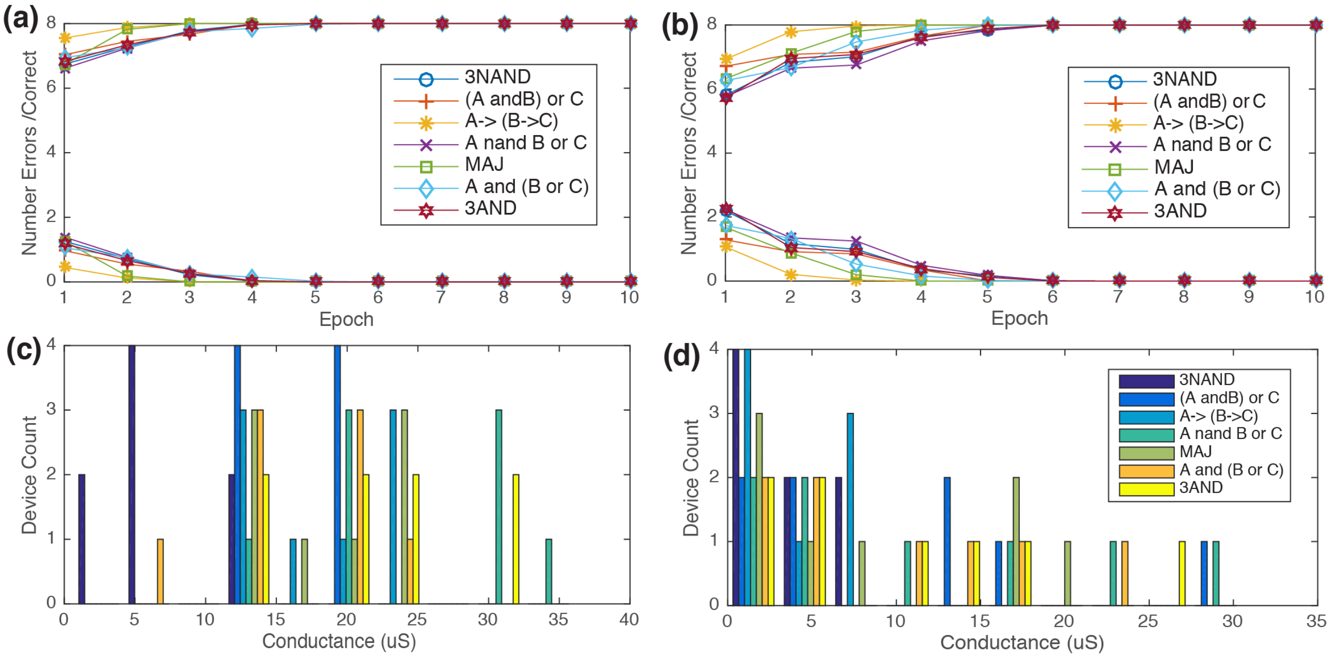

Figure S10. Simulated learning cases using SO (a) and SR (b) programming modes, with uniform nanodevices. 100% of all Monte Carlo iterations for every function learn successfully. Characteristic concluding conductances for one iteration for SO (c) are slightly higher than those for SR (d) since RESET is never used. In every SO case, for every device $\Delta G_+ = 10\% G_{\text{Max}}$; for every SR, $\Delta G_+ = \Delta G_- = 10\% G_{\text{Max}}$. Legend for (c) and (d) is equivalent.

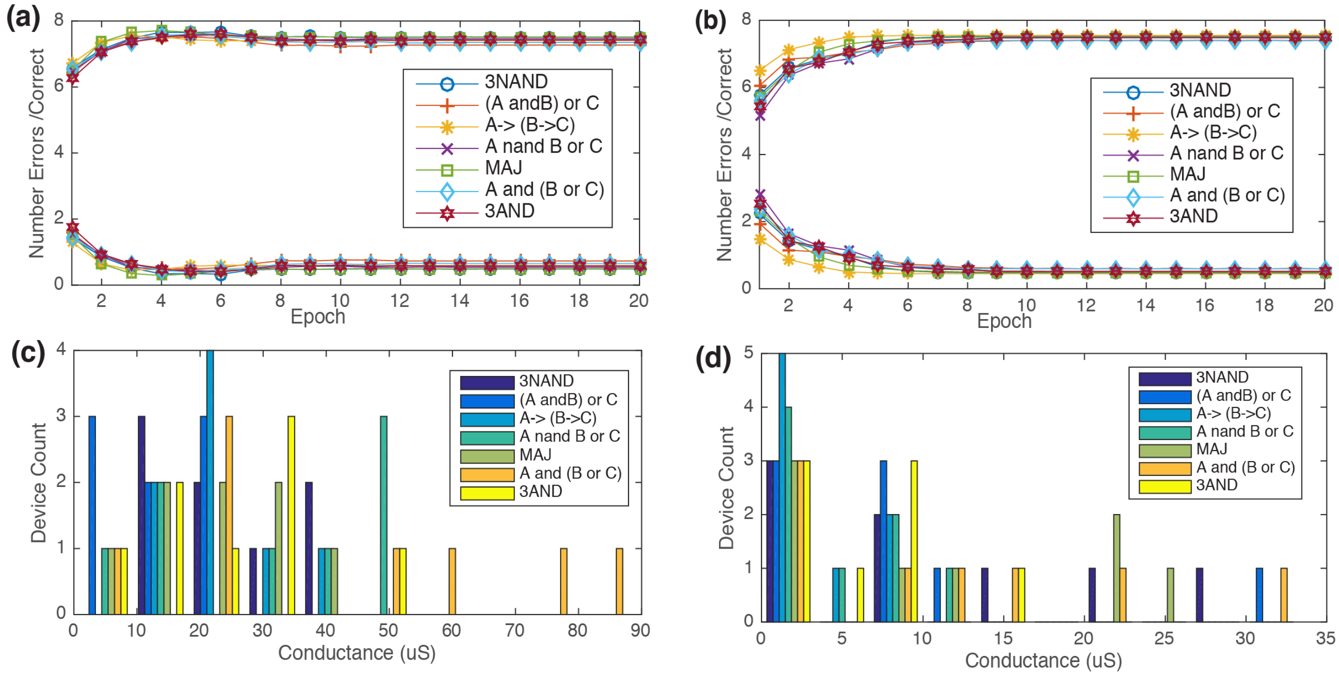

Figure S11. Simulated learning cases using SO (a) and SR (b) learning with variable nanodevices ($\sigma(G_{\mathrm{Max}}) = 40\%$, $\sigma(V_{t1}) = \sigma(V_{t2}) = 10\%$). Learning is imperfect and success varies slightly based on the function. Concluding conductances for a characteristic single iteration (c) are noticeably higher than those for a characteristic SR iteration (d). $\Delta G_+, \Delta G_-$ vary on a device by device basis but always pegged at $10\% G_{\mathrm{Max}}$ for the given device.

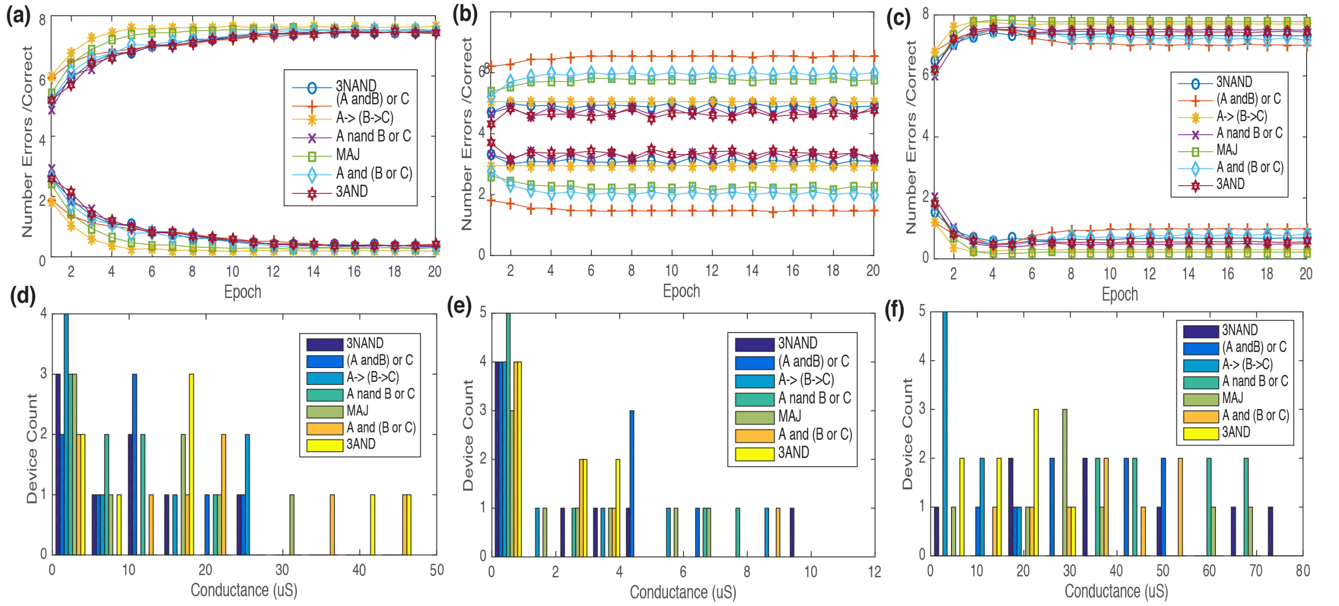

Figure S12. (a) Minor SR asymmetry case ($\Delta G_+ = 15\% G_{\mathrm{Max}}$ $\Delta G_- = 20\% G_{\mathrm{Max}}$) closest to experimental case and listed in Table 2 paper, and characteristic weights (d); (b) SR performance is severely impacted when $\Delta G_+ = 5\% G_{\mathrm{Max}}$ $\Delta G_- = 20\% G_{\mathrm{Max}}$, as concluding weights (e) are uniformly low; (c) Performance is far superior with inverse asymmetry ($\Delta G_+ = 20\% G_{\mathrm{Max}}$ $\Delta G_- = 5\% G_{\mathrm{Max}}$), and weights much higher (f). Device variability same as Fig. S7 in all cases.

b More Complex Tasks

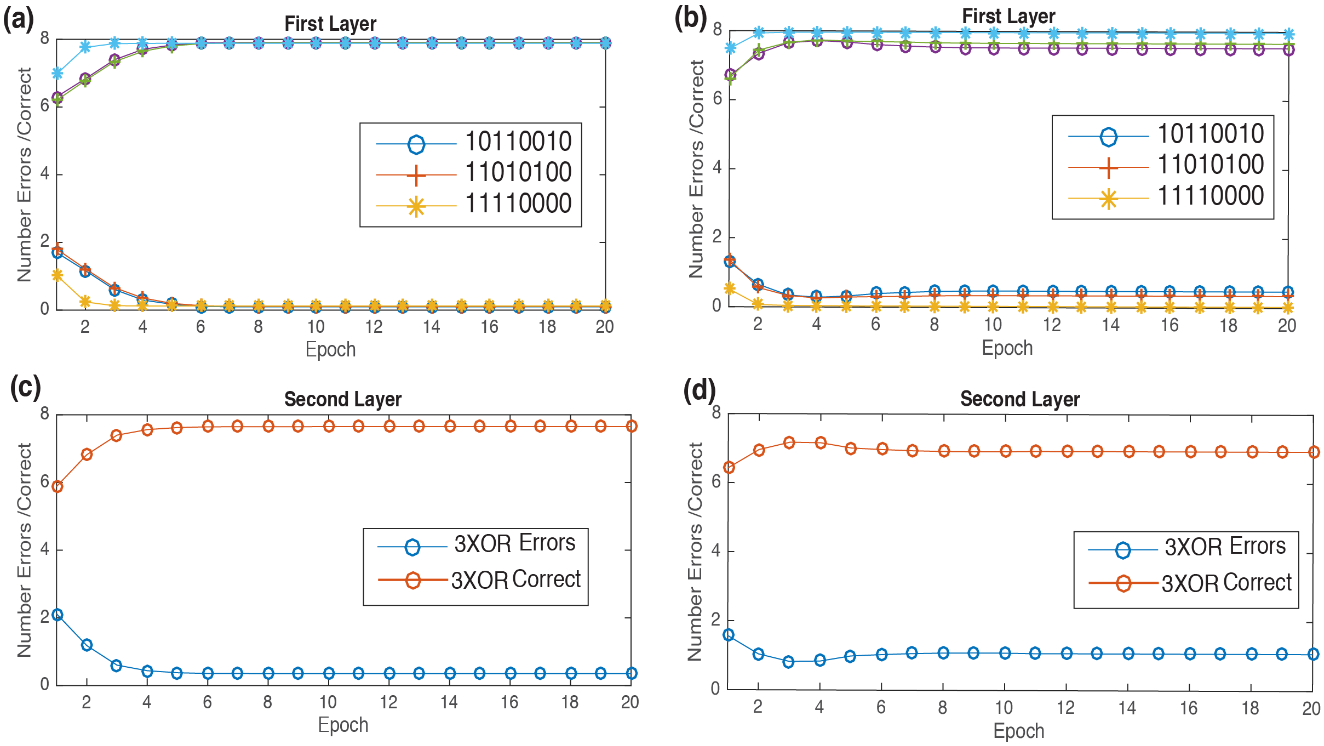

Figure S13. SR mode learning of a non-linearly separable function- the 3XOR function- is depicted in (a) for the composing functions and (c) for the final function. Curves represent mean errors/correct values during each epoch for 500 monte carlo simulations; learning of the first layer functions is nearly perfect, while the second layer function (3XOR) learns in 79.6% cases by mean 30.2 epochs. SO mode learning for the identical task is depicted in (b), (d) for first and second layers respectively. 71% of all cases now learn successfully, at a faster mean 23 epochs. Every simulation assumed device variability at the same levels examined in Figs. S10-S11; $\Delta G_{+,-} = 10\% G_{\text{Max}}$ and the SR case is symmetric at this level.

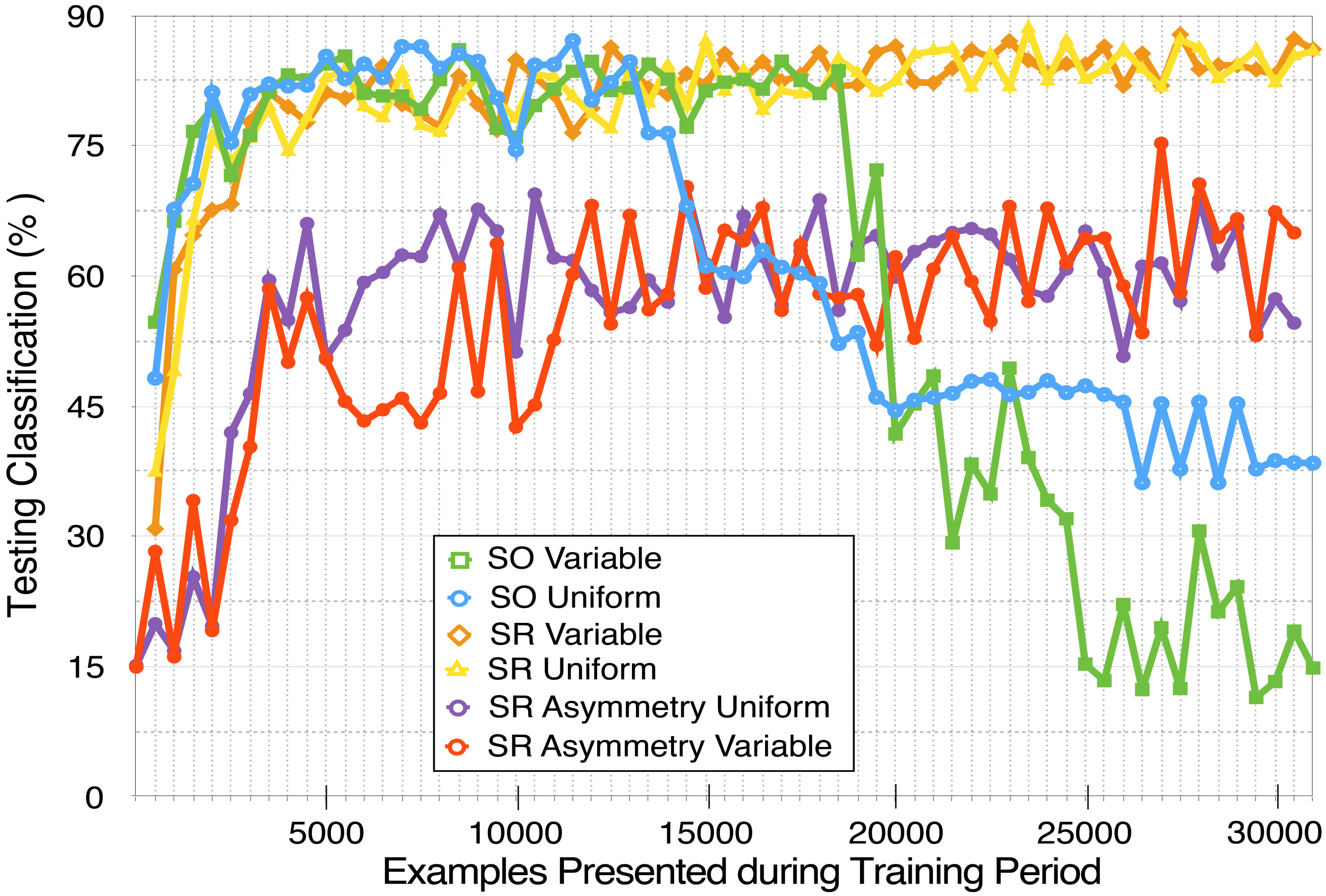

Figure S14. Classification performance as percentage of 10,000 Tests on the MNIST database answered correctly (guess g matches actual class value k) as a function of the number of examples chosen randomly from the pool of training examples (total 60,000) presented to obtain W (weight matrix of all memristive nanodevices). Blue and yellow lines use uniform devices (all have constant thresholds and maximum conductance). Green and orange lines introduce inter-device variability at $\sigma = 10\%$ around characteristic values $\mu(G_{\text{Max}}) = 60uS$, $\mu(V_{\text{t1}}) = 3V$, $\mu(V_{\text{t2}}) = 5.5V$ (for SO). In these cases, nanodevice conductance changes were set as $\Delta G_+ = 5\% G_{\text{Max}}$, $\Delta G_- = 5\% G_{\text{Max}}$ in SR, and $\Delta G_+ = 2.5\% G_{\text{Max}}$ in SO to prevent saturation of weights. Purple (uniform devices) and red (variable) series suggest the case in which every RESET is a violent one: that, is, $\Delta G_+ = 2.5\% G_{\text{Max}}$, $\Delta G_- = 5\% G_{\text{Max}}$. Each point is the average of 5 simulations under different low starting conductances, in the uniform cases, and 10 simulations under different starting and dispersion values, in the variable cases.

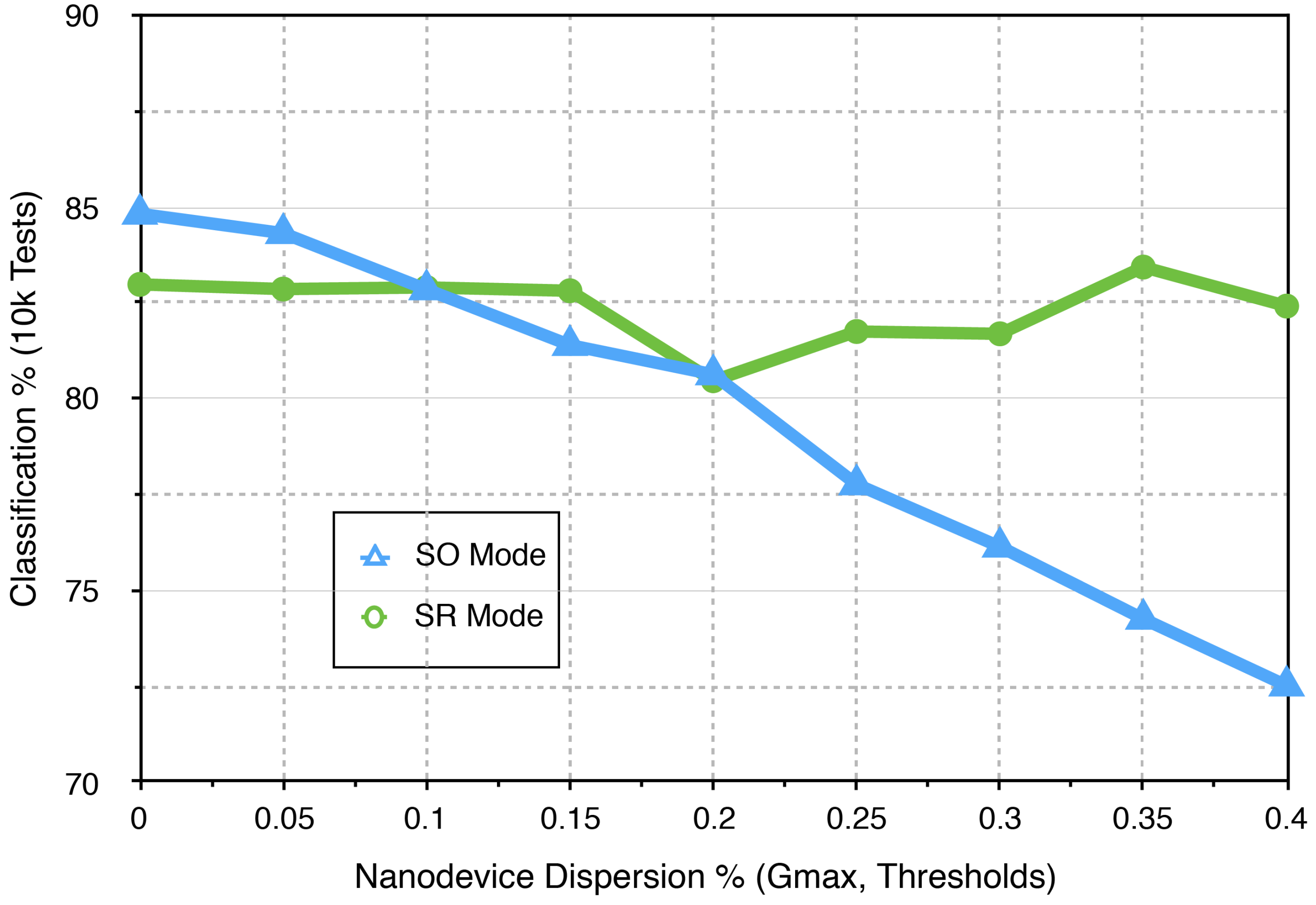

Figure S15. Classification performance (percentage of 10,000 tests answered correctly on the MNIST database) when 5,000 training samples are picked randomly from the training set and iteratively used to set W. Performance is given as the function of increasing dispersion parameter σ used to assign different threshold and maximum conductivity values to each of the simulated 15.6k organic memristive nanodevices for both SO, SR modes along a Gaussian spread. In every case, $\mu(G_{\text{Max}}) = 60\mu S$, $\mu(V_{\text{t1}}) = 3V$, $\mu(V_{\text{t2}}) = 5.5V$ (the last matters only for SR). For each simulation given as a point on the graph, 10 separate simulations were conducted and results averaged to reduce the effect of outliers. As before, $\Delta G_{+} = 5\% G_{\text{Max}}$, $\Delta G_{-} = 5\% G_{\text{Max}}$ for SO, and $\Delta G_{+} = 2.5\% G_{\text{Max}}$.
